# Investigation of Bacterial Infections and Antibiotic Resistance Patterns Among Clinical Isolates in the Center of Iran

**DOI:** 10.1155/ijm/4694690

**Published:** 2025-08-09

**Authors:** Sara Naseri, Maryam Sadeh, Mehdi Fatahi-Bafghi, Mahmood Vakili

**Affiliations:** ^1^Department of Microbiology, Faculty of Medicine, Shahid Sadoughi University of Medical Sciences, Yazd, Iran; ^2^Department of Laboratory Sciences, School of Paramedicine, Shahid Sadoughi University of Medical Sciences, Yazd, Iran; ^3^Department of Community Medicine, Health Monitoring Research Center, Faculty of Medicine, Shahid Sadoughi University of Medical Sciences, Yazd, Iran

**Keywords:** antimicrobial resistance, bacterial infections, D-test, ESBL, molecular identification, MRSA

## Abstract

**Introduction:** Bacterial infection is a considerable problem in hospitals. Thus, this study was executed to appraise the rampancy of bacterial infections, antimicrobial susceptibility patterns, and molecular characterization of isolates among patients in Bafgh Hospital in Yazd, Iran, in 2020.

**Methods:** In the current study, we surveyed 103 isolates of 400 clinical specimens from early March 2020 to September 2020 in Bafgh Hospital. We assessed phenotypic traits and antibiotic resistance with standard microbiological methods. Phenotypic methods were also performed to identify extended-spectrum beta-lactamases (ESBLs) in Gram-negative bacilli, inducible clindamycin resistance, and methicillin resistance in *Staphylococcus* according to CLSI guidelines. Molecular identification of isolates was done by conventional PCR 16S rRNA gene sequencing. Furthermore, we investigated the prevalence of resistant genes including *bla*_TEM_, *bla*_PER-2_, *bla*_CTX-M_, *bla*_SHV_, and *bla*_VEB-1_ in Gram-negative bacteria and the *mec*A gene in staphylococcal species.

**Results:** From 400 different clinical specimens, 103 isolates of Gram-positive and Gram-negative bacteria were isolated. Based on phenotypic and molecular methods, most common isolates were *Escherichia coli* (53 isolates), followed by *Klebsiella* spp. (18 isolates), and *Staphylococcus aureus* (16 isolates). The highest resistance was found in Gram-positive bacteria to erythromycin (66.67%) and penicillin (55.56%), while considering Gram-negative bacteria, the most resistant was cefixime (49.41%) and trimethoprim–sulfamethoxazole (47.05%). In addition, out of 16 *S. aureus* isolates, 62.5% and 17.65% were resistant to methicillin and clindamycin, respectively. Among 83 Gram-negative isolates, 22.89% were ESBL-positive. The prevalence of *bla*_SHV_, *bla*_PER2_, *bla*_TEM_, *bla*_CTX-M_, and *bla*_VEB-1_ genes was 78.31%, 59.03%, 40.96%, 30.12%, and 0%, respectively.

**Conclusions:** The outbreak of bacterial infections is relatively high in hospitals. Recognizing risk agents for bacterial infections and restricting the administration of multidrug-resistant antibiotics is a substantial measure that must be taken to prevent patient mortality.

## 1. Introduction

Bacteria are among the most influential creatures on earth and can be found almost everywhere [1]. Many of them are beneficial for humans [2]. However, some bacteria cause bacterial infections and are known as pathogenic bacteria [2, 3]. Despite global advances in human health, infectious diseases are still a significant challenge [4]. Besides, they are one of the leading causes of death worldwide [5]. Gram-positive bacteria such as *Enterococcus*, *Staphylococcus aureus*, group B *Streptococcus*, and coagulase-negative staphylococci such as *Staphylococcus saprophyticus* and *Staphylococcus epidermidis* are critical human pathogens [6]. For instance, *S. aureus* is an important cause of nosocomial infections. It is among common pathogens, particularly pneumonia, surgical wound and bloodstream infections, skin and soft tissue infections, abscesses, endocarditis, and bacteremia [7, 8]. *S. saprophyticus* plays a significant role as a pathogen in urinary tract infections, especially in young, sexually active women [9]. *Enterococcus* species are also prevalent in bacteremia, endocarditis, urinary tract infections, and central nervous system infections [7]. Important Gram-negative bacteria in infectious diseases are *Pseudomonas aeruginosa* and Enterobacteriaceae, especially *Escherichia coli*, *Citrobacter* spp., *Enterobacter* spp., and *Klebsiella pneumoniae* [10–12]. Among them, *E. coli* has been reported to cause up to 80% of urinary tract infections, especially in women [13, 14]. Most of these bacterial infections can be treated with antibiotics [15]. However, in previous years, excessive and inappropriate use of antibiotics [16, 17] has caused bacteria to become resistant to antibiotics; hence, it has become a global problem [18]. According to reports of CDC's antibiotic resistance threats in the United States in 2019 (2019 AR Threats Report), more than 2.8 million antibiotic-resistant infections occur in the United States each year, leading to the death of more than 35,000 people [19]. Poor infection prevention and control measures can increase the prevalence of antibiotic resistant infections [20]. Methicillin-resistant *S. aureus* (MRSA) was discovered for the first time after identifying methicillin in 1960. Recently, it has been one of the main reasons for bacterial infections in hospitals with a serious challenge of antibiotic resistance [21]. Besides, PBP2a has a very feeble binding tendency to beta-lactams, the principal target of methicillin, and it is encoded by the *mec*A gene [22]. This gene is located on a mobile genetic element called staphylococcal cassette chromosome mec (*SCCmec*). Horizontal interspecific transmission of this element can be an essential factor in the spread of MRSA [23]. Extended-spectrum beta-lactamases (ESBLs) are a group of beta-lactamases, usually resistant to broad-spectrum cephalosporins, penicillins (Ps), and some monobactams [24, 25]. *bla*_TEM_, *bla*_CTX-M_, *bla*_PER_, *bla*_VEB_, and *bla*_SHV_ are the most important beta-lactamases belonging to the Enterobacteriaceae family and are significant agents in multidrug resistance in bacterial infections [26, 27]. Rising antibiotic resistance leads to increased morbidity, higher mortality rates, and substantial costs to global health [28]. Resistance of bacteria to antibiotics is a natural process that cannot be stopped. However, this trend is escalating rapidly due to the unnecessary prescription of antibiotics, prescribing the wrong type, and specifying incorrect durations of use. Therefore, this study was performed to identify bacteria isolated from clinical specimens in Bafgh Hospital in Yazd, Iran, and their antibiotic resistance patterns.

## 2. Materials and Methods

### 2.1. Specimen Collection and Identification

**Figure 1:**
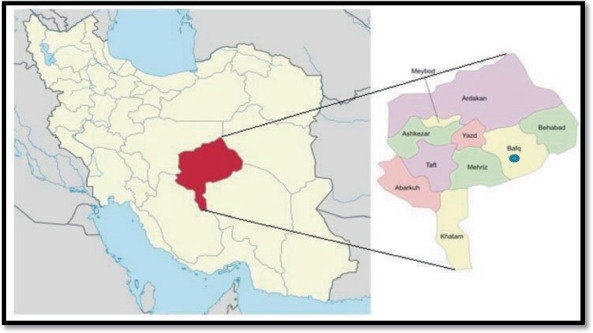
Map of Iran: the location of Bafgh County situated in Yazd Province, Iran (adapted from Hoseini et al. [31]).

In the current study, we surveyed 103 isolates of 400 clinical specimens, including blood (*N* = 6), urine (*N* = 91), wound aspiration (*N* = 4), and stool (*N* = 2), which were isolated by hospital microbiological laboratory from outpatients (*N* = 90) and inpatients (*N* = 13) from early March 2020 to September 2020 in Bafgh Hospital (Figure 1). Initially, each specimen was cultured separately on EMB agar and blood agar in the laboratory. The stool specimens were cultured on an XLD medium and incubated for 24 h at 37°C; then, plates with significant growth were selected for further examination. Overall, 103 positive isolates were obtained from various infections of patients in the hospital. The isolates were transported to TSB medium with 15% glycerol at −20°C to the Faculty of Medicine microbiological laboratory. Subsequently, recultured on blood agar, bacteria were identified using standard microbiological methods, including colony form and Gram staining. Then, differential tests were performed for Gram-positive bacteria including catalase, coagulase, fermentation of mannitol, DNase, susceptibility to novobiocin disks, tolerance to bile esculin, and growth in 6.5% NaCl broth tests [29]. The Gram-negative bacilli were recognized using different culture media, including oxidase, catalase, TSI, LIA, Simon Citrate, urease, SIM, MR-VP, and ONPG disk [30]. It is worth mentioning that this study was considered and approved by the Ethical Committee of Shahid Sadoughi University of Medical Sciences, Yazd, Iran (IR.SSU.MEDICINE.REC.1399.218).

### 2.2. Antimicrobial Susceptibility Testing

After the initial identification of bacterial strains, the disk diffusion (DD) method evaluated antibiotic susceptibility testing of isolated bacteria. Antibiotics that have been used for Gram-positive cocci and Gram-positive bacilli include ciprofloxacin (CP) (5 *μ*m), amikacin (AN) (30 *μ*m), trimethoprim–sulfamethoxazole (SXT) (1.25–23.75 *μ*m), gentamycin (GM) (10 *μ*m), nitrofurantoin^1^ (FM) (300 *μ*m), erythromycin (E) (15 *μ*m), clindamycin (CC) (2 *μ*m), P (10 *μ*m), tetracycline (TE) (30 *μ*m), vancomycin (V) (30 *μ*m) (for the V, determining the minimum inhibitory concentration (MIC) is the standard method, which was not performed in this study). Antibiotics that have been used for Gram-negative bacilli include ampicillin–sulbactam (SAM) (10 *μ*m), SXT (1.25–23.75 *μ*m), AN (30 *μ*m), cefixime (CFM) (5 *μ*m), CP (5 *μ*m), GM (10 *μ*m), ceftriaxone (CRO) (30 *μ*m), cefotaxime (CTX) (30 *μ*m), FM (300 *μ*m), amoxicillin–clavulanic acid (AMC) (30 *μ*m), and meropenem (MEN) (10 *μ*m). Ultimately, the results were interpreted according to CLSI 2021 guidelines [32]. The Gram-positive and Gram-negative bacterial isolates, including *E. coli* (ATCC 25922), *Pseudomonas aeruginosa* (ATCC 27853), and *S. aureus* (ATCC 25923), were used to control the quality of the antibiotic disks according to standard DD test procedures [32].

### 2.3. Phenotypic Detection of Inducible CC Resistance

The D-test method was performed according to CLSI 2021 guidelines [32]. A turbidity matching 0.5 McFarland standard suspension of bacteria was prepared and inoculated on a Mueller–Hinton agar plate. CC (2 *μ*g) and E (15 *μ*g) disks were placed on the medium 15 mm apart. Then, the plates were incubated at 37°C for 24 h. The observation of a D-shaped zone around the CC between the two disks indicates induced resistance to CC [33].

### 2.4. Phenotypic Detection of Methicillin Resistance in *S. aureus*

MRSA was detected by cefoxitin (30 *μ*g) DD test according to CLSI 2021 guidelines [32]. After preparing the bacterial suspension equivalent to the standard 0.5 McFarland and culturing it on the Mueller–Hinton agar plate, the disk was placed on it. Then, the plates were incubated at 37°C for 24 h. Strains with an inhibition zone diameter of ≤ 21 mm were reported as methicillin-resistant staphylococci [34].

### 2.5. Phenotypic Identification of ESBLs

^1^Nitrofurantoin disc is used for urinary tract infections; however, this study used its other clinical and urine specimens.

The phenotypic production of ESBL was recognized in Gram-negative bacteria using the combination disk method, which corresponded to CLSI instructions [35]. A microbial suspension was prepared equivalent to the standard 0.5 McFarland and cultured on Mueller–Hinton agar medium. Subsequently, two disks of ceftazidime (30 *μ*g) and ceftazidime-clavulanic acid (10 *μ*g) were placed at a distance of 20 mm from each other. After 24 h of incubation of the plates at 37°C, observation of a zone of inhibition of ≥ 5 mm around each combination disk was considered positive. *K. pneumoniae* ATCC 700603 and *E. coli* ATCC 25922 were used as positive and negative controls, respectively [36].

### 2.6. DNA Extraction and Genotypic Detection

Genomic DNA extraction was performed using the STET buffer (sodium chloride-TRIS-EDTA-Triton buffer) method [37]. Molecular identification of isolates was performed by PCR method using 16S rRNA gene. Also, for *S. aureus* identification *nuc*A gene was used (primers are mentioned in Table 1). Next, PCR products were examined using 1.5% agarose gel and a 50-bp ladder. Then, intended isolates with a band size of about ~1550 bp were sent for sequencing. Finally, the alignment of sequencing and drawing the phylogenetic tree was examined using JPhydit and MEGA6 software, respectively. *bla*_TEM_, *bla*_PER-2_, *bla*_CTX-M_, *bla*_SHV_, and *bla*_VEB-1_ genes in Gram-negative bacteria and the *mec*A gene in staphylococcal species were amplified by conventional PCR. The primer sequences, PCR conditions, and sizes of the amplified products used in this study are listed in Table 1. The collected data were statistically analyzed using SPSS20 software Version 20, descriptive statistics, and appropriate statistical tests such as chi-square and contingency coefficient. A significance level of 0.05 was considered.

## 3. Results

### 3.1. Characteristics of the Total Patient Population

In this study, from 400 clinical specimens, 103 Gram-positive and Gram-negative bacteria were collected from different age groups, of which 25 (24.27%) and 78 (75.7%) bacteria were isolated from males and women, respectively (Table 2). The specimens contained 88.34% urine, 3.89% wounds, 1.95% stool, and 5.82% blood. The most common section was relevant to outpatients (87.37%), followed by surgical patients (4.85%), CCU patients (2.91%), internal medicine department (2.91%), and pediatric patients (1.94%), respectively.

### 3.2. Results of Phenotypic Tests

Bacteria were identified phenotypically using standard microbiological methods. From 103 isolates, 18 were Gram-positive cocci, 2 were Gram-positive bacilli, and 83 were Gram-negative bacilli (Table 3).

### 3.3. Results of Sequencing

**Figure 2:**
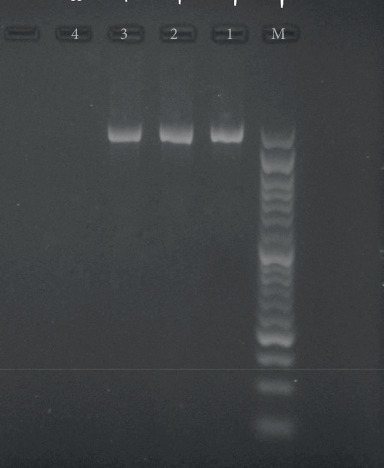
PCR amplification of 16S rRNA gene (~1550 bp). M: ladder 50 bp DNA; 1–3: strains with 16S rRNA gene; 4: negative control.

**Figure 3:**
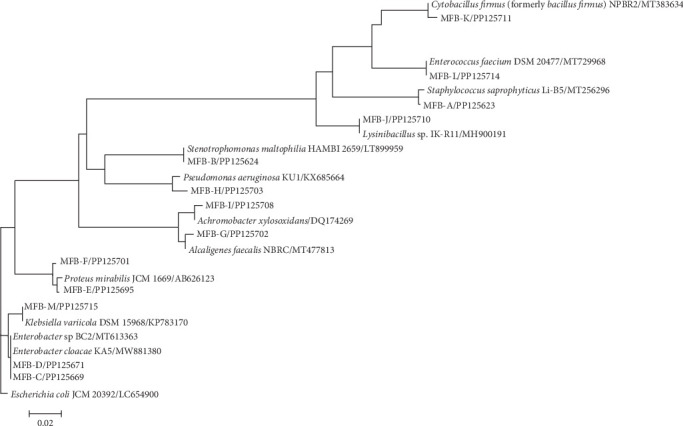
Phylogenetic tree based on 16S rRNA gene sequences.

One isolate was sent for sequencing after identifying bacteria phenotypically from each group with the same phenotypic results (Figure 2). The results showed that there were 18 Gram-positive cocci covering *S. aureus* (16 isolates), *Enterococcus faecium* (1 isolate), *S. saprophyticus* (1 isolate) and 2 Gram-positive bacilli samples, including *Lysinibacillus* spp. (1 isolate) and *Cytobacillus firmus* (1 isolate). Of the 83 Gram-negative isolates, *E. coli* was the most common (53 isolates), followed by *Klebsiella* spp. (18 isolates), *Stenotrophomonas maltophilia* (3 isolates), *Klebsiella aerogenes* (2 isolates), *Enterobacter cloacae* (1 isolate), *Proteus mirabilis* (2 isolates), *P. aeruginosa* (2 isolates) *Alcaligenes faecalis* (1 isolate), and *Achromobacter xylosoxidans* (1 isolate). According to the sequencing results, the phylogenetic tree is shown in Figure 3.

### 3.4. Results of Antimicrobial Susceptibility Testing

**Figure 4:**
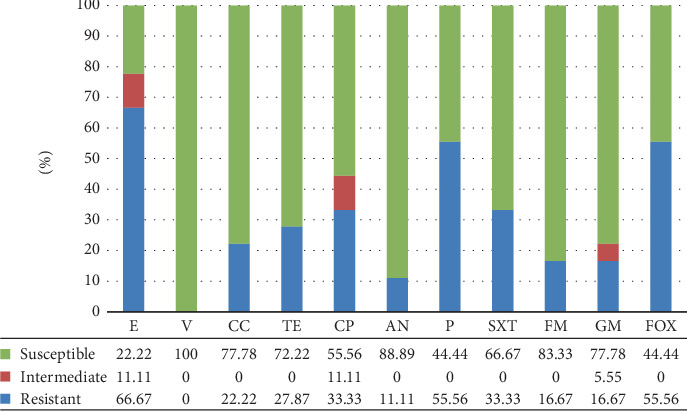
Antimicrobial susceptibility tests in Gram-positive cocci. Erythromycin (E), vancomycin (V), clindamycin (CC), tetracycline (TE), ciprofloxacin (CP), amikacin (AN), penicillin (P), trimethoprime–sulfamethoxazol (SXT), nitrofurantoin (FM), gentamycin (GM), and cefoxitin (FOX).

**Figure 5:**
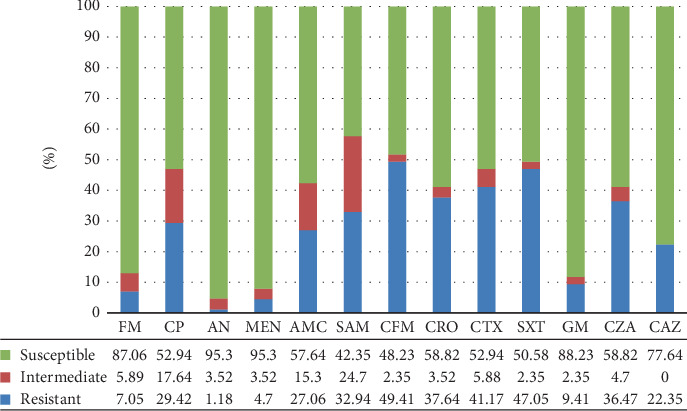
Antimicrobial susceptibility tests in Gram-negative. Nitrofurantoin (FM), ciprofloxacin (CP), amikacin (AN), meropenem (MEN), amoxicillin–clavulanic acid (AMC), amoxicillin–sulbactam (SAM), cefixime (CFM), ceftriaxone (CRO), cefotaxime (CTX), trimethoprim–sulfamethoxazol (SXT), gentamycin (GM), ceftazidime–clavulanic acid (CZA), and ceftazidime (CAZ).

Figure 4 shows the antimicrobial resistance pattern of Gram-positive cocci. The results indicated the highest resistance to E (66.67%) and P (55.56%). In addition, all isolates were sensitive to V, followed by AN (88.8%). Among Gram-negative bacilli, *E. coli* has illustrated the highest resistance to SXT (52.8%) and CFM (50.9%). The most sensitive groups were FM (98.1%), MEN (98.1%), and AN (94.3%). Likewise, *Klebsiella* spp. also displayed the highest resistance rates to CTX (72.2%), SXT (72.2%), CRO (55.5%), and CFM (55.5%), while the most sensitive was reported at 94.4% to MEN and 88.8% to AN. In this study, Gram-negative isolates were extremely resistant to CFM (49.41%) and SXT (47.05%) (Figure 5). Also, two isolates of Gram-positive bacilli were sensitive to all disks except P.

### 3.5. Frequency of MRSA Isolates

The cefoxitin DD test was performed for 16 isolates of *S. aureus*. Findings indicated that 10 isolates (62.5%) (5 women and 5 men) were resistant to methicillin, and six isolates (37.5%) (5 women and 1 man) were sensitive to methicillin. MRSA was observed in urine (5 isolates), blood (3 isolates), and wound (2 isolates) specimens.

### 3.6. Frequency of Inducible CC-Resistant Isolates

All 17 isolates of *Staphylococcus* (16 *S. aureus* and 1 *S. saprophyticus*) were investigated. Three isolates (three men) were identified with inducible CC resistance (17.65%). These three isolates were isolated from urine, blood, and wound specimens.

### 3.7. Prevalence of ESBL in Gram-Negative Specimens

Among the 83 Gram-negative isolates detected by the combination disk method, 19 strains (22.89%) demonstrated the ESBL phenotype, and 64 strains (77.10%) were ESBL negative. The ESBL producers were mostly from urine isolates (*n* = 17; 89.4%) and some other specimens, including wounds (*n* = 1; 5.2%) and blood (*n* = 1; 5.2%).

### 3.8. Identification of the *nuc*A Gene by PCR

**Figure 6:**
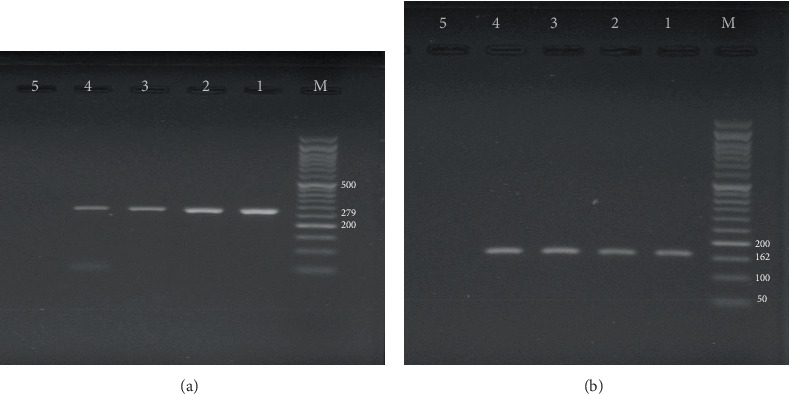
(a) Genotypic recognition of *nuc*A gene. M: ladder 50 bp DNA; 1: positive control (279 bp); 2–4: *nuc*A positive isolates; 5: negative control. (b) Genotypic recognition of *mec*A gene. M: ladder 50 bp DNA; 1: positive control (162 bp); 2–4: *mec*A positive isolates; 5: negative control.

The diagnosis of *S. aureus* among 17 isolates of *Staphylococcus* spp. was done by PCR using the *nuc*A gene. The result showed that all 16 isolates were positive for the *nuc*A gene (Figure 6).

### 3.9. Prevalence of *mec*A Gene in *Staphylococcus* spp.

The frequency of the *mec*A gene among 17 isolates of *Staphylococcus* was appraised, and 10 isolates were found to be positive for *mec*A (Figure 6).

### 3.10. Molecular Result of ESBL Producer Genes in Gram-Negative Bacteria

**Figure 7:**
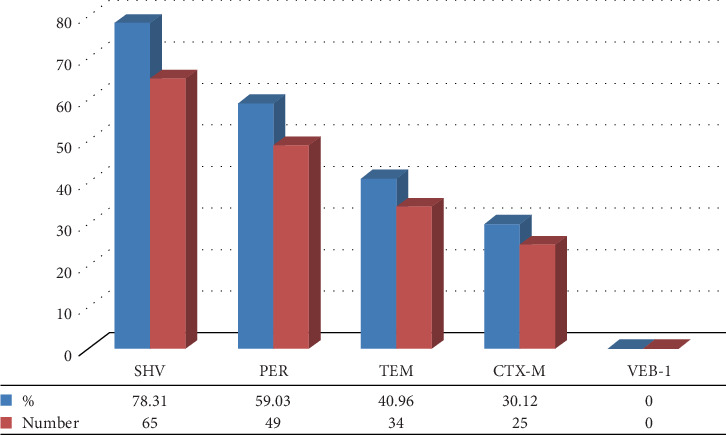
Outbreak of ESBL producing genes in all Gram-negative bacteria.

**Figure 8:**
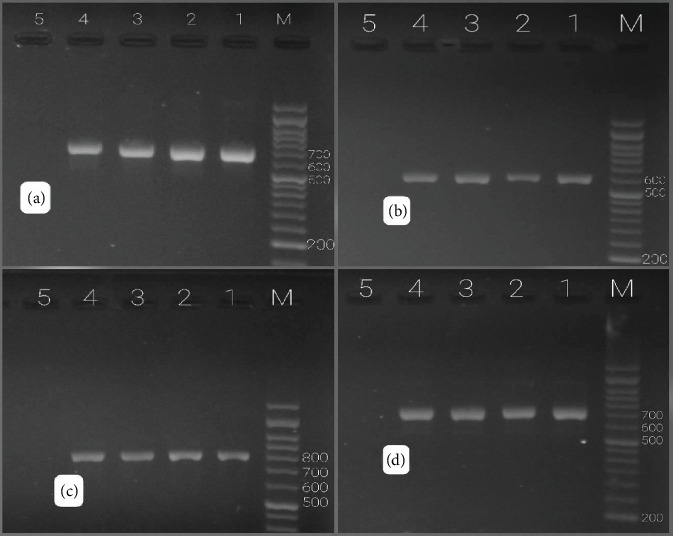
(a) *bla*_TEM_ gene (692 bp). M: ladder 50 bp DNA; 1: positive control; 2–4: *bla*_TEM_ positive isolates; 5: negative control. (b) *bla*_CTX-M_ gene (585 bp). M: ladder 50 bp DNA; 1: positive control; 2–4: *bla*_CTX-M_ positive isolates; 5: negative control. (c) *bla*_SHV_ gene (795 bp). M: ladder 50 bp DNA; 1: positive control; 2–4: *bla*” positive isolates; 5: negative control. (d) *bla*_PER-2_ gene (739 bp). M: ladder 50 bp DNA; 1: positive control; 2–4: *bla*_PER-2_ positive isolates; 5: negative control.

**Figure 9:**
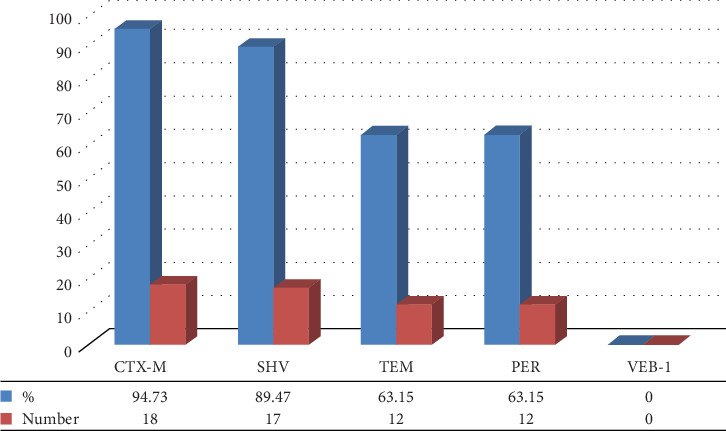
Distribution of ESBL producer genes in ESBL-positive strains of Gram-negative bacteria.

Of 83 Gram-negative bacteria, 69 had one or more ESBL genes. The results of the ESBL-producing genes outbreak showed that *bla*_SHV_, *bla*_PER2_, *bla*_TEM_, *bla*_CTX-M_, and *bla*_VEB-1_ genes were 78.31%, 59.03%, 40.96%, 30.12%, and 0%, respectively (Figures 7 and 8). Among 19 ESBL-positive strains of Gram-negative bacteria, *bla*SHV (89.47%) had the highest percentage of prevalence, followed by *bla*_PER_ (63.15%), *bla*_TEM_ (63.15%), *bla*_CTX-M_ (94.73%), and *bla*_VEB-1_ (0) (Figures 8 and 9; Table 4). Among 19 ESBL-positive strains, 16 isolates were related to *E. coli*, and 3 isolates were related to *Klebsiella* spp. The urine specimens contained the most genes (*n* = 17, 89.47%), followed by blood (*n* = 1, 5.26%) and wound (*n* = 1, 5.26%).

## 4. Discussion

Recently, due to the increasing rampancy of antibiotic resistance, continuous mutations can be observed in infectious diseases [46]. Statistical results illustrated that not taking efficient measures to prohibit them will cause the death of 10 million people due to antimicrobial resistance till 2050 [47]. In the last few years, most studies have been conducted on the most influential hospital strains, including *E. coli*, *Salmonella*, and *S. aureus*, focusing on their increasing trend toward antibiotic resistance [48, 49]. For instance, the production of ESBLs has led to resistance to various kinds of beta-lactam antibiotics [50]. The most prevalent of these genes are *bla*_TEM_, *bla*_SHV_, and *bla*_CTX-M_ [51]. In some MRSA isolates, PBP2a (the *mec*A P-binding protein) is responsible for resistance to methicillin and all beta-lactam antibiotics [52]. In this study, 400 different clinical specimens from blood, urine, wound, and respiratory secretions were separated from outpatients and inpatients in Bafgh-Yazd Hospital from March 2020 to September 2020. One hundred and three isolates were positive culture; 24.27% belonged to male patients, and 75.7% to female patients. In the present study, the highest incidence of hospital-acquired bacteria was observed in people between 1 and 15 years of age (28.84%). Most isolates were detected in outpatients (87.37%), and the most common type of isolates was related to urine (88.34%). From 103 positive isolates, 83 Gram-negative bacilli and 20 Gram-positive bacteria were identified. The most common isolates were *E. coli* (53 isolates), followed by *Klebsiella* spp. (18 isolates), *S. aureus* (16 isolates), *S. maltophilia* (3 isolates), *K. aerogenes* (2 isolates) (this species, previously explained as *Enterobacter aerogenes*, is an opportunistic pathogen associated with infections containing pneumonia, sepsis, and urinary tract infection [53, 54]), *P.mirabilis* (2 isolates), and *P. aeruginosa* (2 isolates); *E. faecium*, *E. cloacae*, *A. faecalis*, *A. xylosoxidans*, *S. saprophyticus*, *Lysinibacillus* spp., and *Cytobacillus firmus* each had only one isolate. No significant relationship was observed between age, gender and the prevalence of hospital bacteria (*p* > 0.05). In a study conducted at Hiwot Fana specialized hospital in eastern Ethiopia in 2017, 394 patients were investigated, among which 56.6% were females and 43.4% were males. Fifty four isolates were positive culture. Most specimens were collected from the gynecology and obstetrics wards (26.1%). It was found that *S. aureus* (18.5%) was the most common isolate, followed by *E. coli* (16.7%) [55]. The prevalence of bacteria in this study was slightly different from ours, probably due to the sample size and different hospital departments. Similar to the present study results, Azari et al. conducted research in Iran, in which 96 Gram-negative isolates were collected during 3 months (from March 2017 to May 2017). The most prevalent isolates were as follows: *E. coli* (88.5%), *K.pneumoniae* (5.2%), *E. cloacae* (4.2%), and *P. aeruginosa* (2.1%), respectively [56]. Another similar study was conducted in eastern Uganda, and 3092 were found from two microbiology laboratories from January 2016 to December 2018. The most prevalent bacteria were *E. coli* (*n* = 442; 33.8%), *S.aureus* (*n* = 376; 22.8%), *K. pneumoniae* (*n* = 237; 18.2%), and *Streptococcus pneumoniae* (*n* = 76; 5.4%) [57]. In our study, in Gram-positive bacteria, the highest resistance was observed in E (66.67%), P (55.56%), and cefoxitin (55.56%). Also, all strains were sensitive to V. In addition, all 16 *S. aureus* isolates had 62.5% resistance to methicillin and 17.65% induction resistance to CC. In Gram-negative bacteria, the isolates were highly resistant to CFM (49.41%) and SXT (47.05%) and were least susceptible to AN (95.3%) and MEN (91.77%). Among 83 Gram-negative strains, 22.89% were positive for ESBL. Like our result, Tolera et al. reported that *S. aureus* showed 80% resistance to chloramphenicol and E. They also reported that MRSA comprises 88.9% of all *S. aureus* isolates [55]. In another study by Ahani Azari et al., *E. coli* had the highest resistance to ampicillin (89.4%), cephalothin (76.4%), and FM (75.2%) and the lowest resistance to norfloxacin (30.5%) [56]. Obakiro et al. reported high rates of antimicrobial resistance to cephalosporins (CTX), Ps (ampicillin), GM, and cotrimoxazole and relatively lower resistance to imipenem, V (for *S. aureus*), and piperacillin [57]. A study conducted in central Uganda also identified high resistance rates among *E. coli* and *K. pneumoniae* isolates combined: SXT (70%), amoxicillin–clavulanate (36%), piperacillin (27%), cefoxitin (22%), cefepime (15%), chloramphenicol (20%), CP (11%), and GM (11%) [58]. In another similar study in Iran, 65 isolates were isolated from urine, feces, blood, sputum, and wound. Of 52 Gram-negative isolates, 9.6% of strains were ESBL positive; and among 8 *S. aureus*, six were methicillin resistant [59]. In a similar study but with a larger sample size in Central Hospital IPS, 145 *S. aureus* isolates were collected that caused skin, soft tissue, and osteoarticular infections in pediatric patients. The resistance to methicillin and CC was 67% and 13%, respectively [60]. In another study with 185 Gram-negative bacteria, the prevalence of ESBL positive was found to be 85.8% [61]. In the present study, the frequency of *nuc*A and *mec*A genes among 17 species of *Staphylococcus* isolated from clinical specimens showed that 16 isolates were positive for *nuc*A, and 10 isolates were positive for *mec*A. Rajabiani et al. also conducted a study in Iran, and it was reported that 92 (36/8%) out of 250 isolates of *S. aureus* carried the *mec*A gene [62]. In a study conducted on patients in the burn center of a hospital in Iran, the prevalence of the *mec*A gene was reported to be 87.36% [63]. From January 2017 to December 2018, Jyothi and Metri in India collected 45 *S. aureus* to detect the *mec*A gene using PCR and found 20 MRSA isolates and 25 MSSA isolates [64]. In this study, the results of ESBL producing genes outbreak showed that *bla*_SHV_, *bla*_PER_, *bla*_TEM_, *bla*_CTX-M_, and *bla*_VEB-1_ genes were 78.31%, 59.03%, 40.96%, 30.12%, and 0%, respectively. From 2010 to 2015, Dan Li et al. isolated 207 *E. coli* from bloodstream infection specimens in China. The results of ESBL genes were reported as follows: 0.48% (*bla*_KPC_), 57% (*bla*_TEM_), 23.67% (*bla*_CTX-M-1_), 18.84% (*bla*_CTX-M-9_), and 1.93% (*bla*_SHV_) [65].

In our study, the *bla*_SHV_ gene is the most prevalent, but in the mentioned study, the *bla*_TEM_ gene was the most prevalent. Unlike the results of the present study, from January 2016 to February 2017, Ghane and Adham collected 972 urine specimens in Iran. Five hundred cases were culture-positive for *E. coli*. Among the ESBL-positive isolates, the *bla*_TEM_ gene was reported to be 44.72% (*n* = 85), while the *bla*_PER_ gene was not found in any of these isolates [66].

## 5. Conclusion

Urinary tract infections are the most common type of bacterial infection. The most common bacteria causing these infections are *E. coli*. The pathogenesis of UTI begins with the contamination and colonization of the urinary tract area. The bladder epithelium is attacked, and the released toxins damage the host tissue, resulting in a urinary infection. Severe cases can also lead to life-threatening diseases such as bacteremia, septicemia, and urosepsis. High resistance among *E. coli* isolates was reported to CFM and SXT. Hence, it is suggested that these antibiotics not be used as first-line medication. Additionally, it is essential to identify the significant factors in bacterial infections and determine the pattern of antibiotic resistance to prevent and control the spread of factors causing antibiotic resistance.

## Figures and Tables

**Table 1:** Nucleotide sequence of the primers and conditions used for conventional PCR of genes in this study.

**Gene**	**Primer (5**⁣′**-3**⁣′**)**	**Amplicon size (bp)**	**PCR conditions**	**Ref.**
27F 1525R	5⁣′-AGAGTTTGATCCTGGCTCAG-3⁣′ 5⁣′-TGCACACAGGCCACAAGGGA-3⁣′	~1550	94°C, 5 min; 35 cycles of 94°C, 1 min; 64°C, 1 min; 72°C, 2 min; 72°C, 10 min	[38]
*nuc*A	5⁣′-GCGATTGATGGTGATACGGTT-3⁣′ 5⁣′-AGCCAAGCCTTGACGAACTAAAGC-3⁣′	279	94°C, 3 min; 37 cycles of 95°C, 1 min; 60°C, 45 s; 72°C, 1 min; 72°C, 10 min	[39]
_*mec*_A	5⁣′-TCCAGATTACAACTTCACCAGG -3⁣′ 5⁣′-CCACTTCATATCTTGTAACG-3⁣′	162	94°C, 3 min; 40 cycles of 94°C, 30s; 55°C, 30 s; 72°C, 1 min; 72°C, 5 min	[40]
*bla*_TEM_	5⁣′-ATCAGCAATAAACCAGC-3⁣′ 5⁣′-CCCCGAAGAACGTTTTC-3⁣′	692	95°C, 1/20 min; 33 cycles of 95°C, 1 min; 62°C, 15 s; 72°C, 1 min; 72°C, 30s	[41]
*bla*_PER-2_	5⁣′-CGCTTCTGCTCTGCTGAT-3⁣′ 5⁣′-GGCAG CTTCTTTAACGCC-3	739	94 °C, 5 min; 30 cycles of 94°C, 1 min; 54°C, 15 s; 72°C, 1 min; 72°C, 5 min	[42]
*bla*_CTX-M_	5⁣′-CGCTTTGCGATGTGCAG-3⁣′ 5⁣′-ACCGCGATATCGTTGGT-3⁣′	585	94 °C, 5 min; 28 cycles of 95°C, 1 min; 60°C, 1 min; 72°C, 1 min; 72°C, 5 min	[43]
*bla*_SHV_	5⁣′-AGGATTGACTGCCTTTTTG-3⁣′ 5⁣′ ATTTGCTGATTTCGCTCG-3⁣′	795	95°C, 3 min; 33 cycles of 94°C, 1 min; 50°C, 30 s; 72°C, 1 min; 72°C, 5 min	[44]
*bla*_VEB-1_	5⁣′-ACGAAGAACAAATGCACAAGG-3⁣′ 5⁣′- GAACAGAATCAGTTCCTCCG-3	375	94 °C, 5 min; 28 cycles of 94°C, 1 min; 55°C, 30 s; 72°C, 1 min; 72°C, 5 min	[45]

**Table 2:** Age group distribution of patients.

**Sex**	**Under 1 year (%)**	**1–15 (%)**	**16–30 (%)**	**31–50 (%)**	**51–70 (%)**	**Over 70 years (%)**	**Total (%)**
Female	7 (6.79)	26 (25.24)	22 (21.35)	13 (12.62)	6 (5.82)	4 (3.88)	78 (75.7)
Male	5 (4.85)	2 (1.94)	3 (2.91)	4 (3.88)	2 (1.94)	9 (8.73)	25 (24.27)

**Table 3:** The results of phenotypic identification of bacteria in this study.

	**Bacteria**	**Number (%)**
Gram-positive bacteria	*Staphylococcus aureus*	16 (15/53)
	*Staphylococcus saprophyticus*	1 (0/97)
	*Enterococcus* spp.	1 (0/97)
	*Bacillus*	2 (1/94)

Gram-negative bacteria	*E. coli*	53 (51/45)
	*Klebsiella* spp.	18 (17/47)
	*Enterobacter* spp.	3 (2/91)
	*Proteus mirabilis*	2 (1/94)
	*Pseudomonas aeruginosa*	2 (1/94)
	Other Gram-negative bacilli	5 (4/85)

**Table 4:** Outbreak of *β*-lactamase coding genes *bla*TEM, *bla*SHV, *bla*CTX-M, *bla*PER2, and *bla*VEB-1 in ESBL positive strains.

**Number**	***bla*_TEM_**	***bla*_CTX-M_**	***bla*_VEB-1_**	***bla*_SHV_**	***bla*_PER-2_**
1	−	**+**	−	**+**	−
2	**+**	**+**	−	**+**	**+**
3	**+**	**+**	−	**+**	**+**
4	−	**+**	−	**+**	−
5	−	**+**	−	**+**	**+**
6	**+**	**+**	−	**+**	**+**
7	**+**	**+**	−	**+**	−
8	−	−	−	−	−
9	−	**+**	−	**+**	**+**
10	**+**	**+**	−	**+**	**+**
11	−	**+**	−	**+**	**+**
12	**+**	**+**	−	**+**	**+**
13	**+**	**+**	−	**+**	**+**
14	−	**+**	−	**+**	**+**
15	**+**	**+**	−	**+**	**+**
16	**+**	**+**	−	**+**	−
17	**+**	**+**	−	**+**	−
18	**+**	**+**	−	−	−
19	**+**	**+**	−	**+**	**+**

## Data Availability

The data that supports the findings of this study are available in the supporting information of this article.
